# Sustainable Health-Related Quality of Life in Older Adults as Supported by the vINCI Technology

**DOI:** 10.3390/s23042287

**Published:** 2023-02-18

**Authors:** Ciprian Dobre, Lidia Băjenaru, Rozeta Drăghici, Gabriel-Ioan Prada, Alexandru Balog, Anna Marie Herghelegiu

**Affiliations:** 1 Faculty of Automatic Control and Computers, University Politehnica of Bucharest, 060042 Bucharest, Romania; 2 National Institute for Research and Development in Informatics, 011455 Bucharest, Romania; 3 “Ana Aslan” National Institute of Gerontology and Geriatrics, 011241 Bucharest, Romania; 4 Department of Geriatrics and Gerontology, “Carol Davila” Universty of Medicine and Pharmacy, 050474 Bucharest, Romania; 5 Doctoral School of Economic Informatics, Bucharest University of Economics Studies, 010374 Bucharest, Romania

**Keywords:** health-related quality of life, older adults, vINCI technology, ambient assisted living, technology for sustainable health, physical activity

## Abstract

The vINCI technology represents an innovative instrument developed specifically but not exclusively for older adults by technology researchers together with a medical team specialized in geriatrics and gerontology. It was designed to be independently and effortlessly used by older adults in the comfort and safety of their own environment. It is a modular and flexible platform that can integrate a large array of various sensors and can easily adapt to specific healthcare needs. The pilot study tested sensors and standardized instruments capable of evaluating several care-related parameters and of generating personalized feedback for the user dedicated to optimizing physical activity level, social interaction, and health-related quality of life. Moreover, the system was able to detect and signal events and health-related aspects that would require medical assistance. This paper presents how the innovative vINCI technology improves quality of life in older adults. This is evidenced by the results obtained following the clinical validation of the vINCI technology by older adults admitted to the Ana Aslan National Institute of Gerontology and Geriatrics (NIGG) in Bucharest.

## 1. Introduction

Human communities and medical systems worldwide have been facing the new reality characterized by many consequences of an ageing population. During the next few decades, by 2050, almost one-quarter of the world’s population will be 60 years of age and older [1]. In the European Union, the disability-free life expectancy (healthy life years) at the age of 65 is approximately 10 years for both women and men [2]. The goal of medical–social interventions is not only to increase longevity but also to maintain independent functioning, active contribution to society, and a good health-related quality of life in older age. Cerebrovascular, heart diseases, and cancer are the main causes of death and they all have in common a series of modifiable lifestyle risk factors such as poor diet, tobacco use, alcohol consumption, and physical inactivity [3]. These factors along with social isolation, depression, cognitive impairment, physical disabilities, sensory impairments, and lack of access to specialized medical care have a significant negative impact on well-being and quality of life in older age. Present medical and social care practices are based on a passive approach where services are provided when requested. Arguably, this archetype suggests issues that can raise some debate. Even though scientifically supported and overall cost-effective, preventive medicine, a more proactive approach, is not a consistent practice and plays only a minor role within healthcare systems [4,5]. The negative effects of risk factors are dose- and exposure-duration-dependent across the lifespan. It has been established that especially older people can greatly benefit from risk reduction; however, behavior modification is difficult to achieve and it is a complex process [4]. When successfully implemented, preventive interventions require a great deal of time and human resources and, in the specific context of the older population, more specialized trained healthcare professionals [6,7,8,9]. Caring for seniors with various degrees of disabilities, either physical or cognitive, represents a constant challenge for families and communities. When displaced from their homes in order to be placed in long-term care facilities, their well-being and health status often significantly decline. The desirable approach is to provide assistance and care at home, thus enabling older people to live independently and, of course, focusing specialized health care on maintaining functional independence for the longest possible time.

Nowadays there are opportunities for meeting the care needs of older adults. In this respect, more and more research efforts and successful results have led to reshaping the technology landscape with increasingly advanced technological solutions such as biosensors, ambient sensors, and artificial intelligence [10]. Widespread availability of wireless devices and high-speed internet access technologies has also contributed to augment the care and assistance available for seniors [11]. Any technological solution for older people has to be adapted to their characteristics, such as visual or hearing impairments, cognitive function status, level of education, computer literacy, and financial resources. The vINCI technology represents an innovative instrument developed, specifically but not exclusively, for seniors, by computer sciences researchers together with a medical team specialized in geriatrics and gerontology. It was designed to be independently and effortlessly used by seniors in the comfort of their own environment. It is a modular and flexible platform that can integrate a large array of various sensors and therefore easily adapts to specific care needs. The pilot study tested a system consisting of sensors and standardized instruments capable of evaluating several parameters and also of generating a personalized feedback for the user dedicated to optimizing physical activity level, social interaction, and health-related quality of life. Moreover, the system was able to detect and signal events and health-related aspects that would require medical assistance [12,13].

In this paper, we present the results of the pilot vINCI technology on health-related quality of life (QoL), which was tested on an adequate sample of older adults. The system integrates smart devices with monitoring sensors for older adults to collect physical activity, psychological, and social parameters, as well as health and context information describing each subject.

### 1.1. Health-Related Quality of Life

Many authors agree that when defining QoL there are two main domains: objective domains (such as health status, economic status, social functioning and status, housing, etc.) and subjective domains (emotional and psychological well-being, personal fulfillment, morale, self-esteem, etc.); all these should be accounted for [14,15]. Another discussion of these concepts entails the argument of imposed values of life as opposed to self-reported dimensions of quality of life. The World Health Organization (WHO) defines QoL as “an individual’s perception of their position in life in the context of the culture and value systems in which they live and in relation to their goals, expectations, standards and concerns” [16].

The concept of health-related quality of life, as defined by the WHO, introduces a link between two relevant terms: quality of life and health. In 1948, the WHO formulated the definition of health as follows: “a state of complete physical, mental and social well-being and not merely the absence of disease or infirmity”. There are two aspects that need to be taken into account when evaluating the health-related quality of life: the approach should be multidimensional, which means that is important to know the physical, social, and psychological characteristics of the individual and it should be monitored from both perspectives (objective and subjective) for each domain [17,18,19,20,21,22].

In older age, health inevitably becomes the main concern of an individual and has the most important load on a person’s QoL and well-being [23,24]. Physical activity is a major determinant of health-related self-perceived quality of life, especially in older age compared to younger adults [25,26].

The World Health Organization Quality of Life Questionnaire—Short Form (WHOQOL-BREF) is a “generic” instrument, centered on the self-reported impact of health status on social activities, psychological well-being, and autonomy. The WHOQOL-BREF instrument has been extensively used in medical and social research and public policy-making, and has been successfully applied in clinical studies on older populations [27].

### 1.2. vINCI Technology

The vINCI project (Clinically-validated INtegrated Support for Assistive Care and Lifestyle Improvement: the Human Link) has developed an assistive monitoring technology for the elderly. vINCI, an integrated and validated framework, using the internet of things (IoT), provides monitoring services and nonintrusive assistance to the elderly, to support them but also their families, who do not have the time or opportunity to care for them permanently. The vINCI system is also aimed at people who provide care and medical assistance to the elderly, as well as various medical organizations. The challenge for vINCI technologies is to meet the needs of older adults, adapt to cognitive and perceptual decline, and support older adults in daily activities while protecting privacy, independence, and information security.

The monitored person downloads a free application on their mobile phone. Using the application on the smart phone, or the web version of vINCI technology, the user creates an account and gives the application access to personal data. From the mobile application, the person can periodically fill in a series of medical questionnaires to which otherwise he/she would have had access only after complex medical consultations: a questionnaire to identify the perception of quality of life, a questionnaire to identify the perception of physical activity, and another to rate their level of physical activity and mental comfort. The application also provides the scores obtained, even in time, for the respective questionnaires. From the dashboard (web version), the family can track in real time the monitored parameters of the person.

vINCI is a clinically validated ambient intelligence framework, where multiple wearable and ambient devices work together to create an aggregated solution able to capture the various facets of events leading to the decrease in the perceived quality of life as associated with old age.

The vINCI application connects several innovative user-oriented devices: a smart watch (Fitbit and CMD watch, provided by the partner Connected Medical Devices, Bucharest, Romania), smart insoles, and tablets on which users can answer surveys and questions about their mood. All data is aggregated and analyzed by a phone application (vINCI app, which the user uses) and a web platform (vINCI dashboard, used by the owners). This application intends to create a much clearer picture of the patient’s health, life, and activities, and also provide intelligent care for the elderly in clinics. The final goal is to use the data collected from devices, analyze it, and use the results to improve the quality of life of the monitored patients and increase the active ageing rate. The technology designed for user interaction was developed with the active involvement of the elderly [13,28,29].

vINCI technology was tested in the pilots proposed in the project. The most relevant was the pilot developed at the Ana Aslan National Institute of Gerontology and Geriatrics (NIGG), Bucharest, Romania, were there was a salon dedicated to the project and medical staff to validate the technology with patients [12].

In parallel, a second medical pilot was deployed in Cyprus, in an institute working with the elderly belonging to the University of Nicosia (medical partner).

In previously published papers, important research was presented on technology components in various stages of development. Preliminary versions of the vINCI platform, as well as the involvement of patients and their caregivers in the design of the technology, are demonstrated in the work [29]. This consists of investigating their needs and requirements from information collected using the specific questionnaires.

All collected data are integrated into a model that ontologically models the medico-geriatric profile of a patient. This innovative model was developed following an extensive study that followed both medical records in the field and consulted specialists in various relevant fields. Finally, the model was tested and then implemented via a single service on the vINCI platform, where all data are aggregated unitarily for a patient. Two things were accomplished in this patient model: the data obtained in the tests were used for medical validation of the evidence obtained by monitoring with the help of the technology, and an algorithm was proposed to interpret the monitoring information (providing the score that the user receives via the app) [30]. Preliminary study of the establishment of the quality of life model proposed in the project and analysis methods are presented in detail in [31,32]. An original analysis of the data collected through the WHOQoL-BREF questionnaires from the elderly, which identifies groups with distinct QOL profiles, is presented in [33].

### 1.3. vINCI Architecture

The vINCI system, an application that offers non-intrusive monitoring and care to the elderly, contributes to improving quality of life (QoL) by offering modern and efficient solutions that meet their expectations [12].

**Figure 1:**
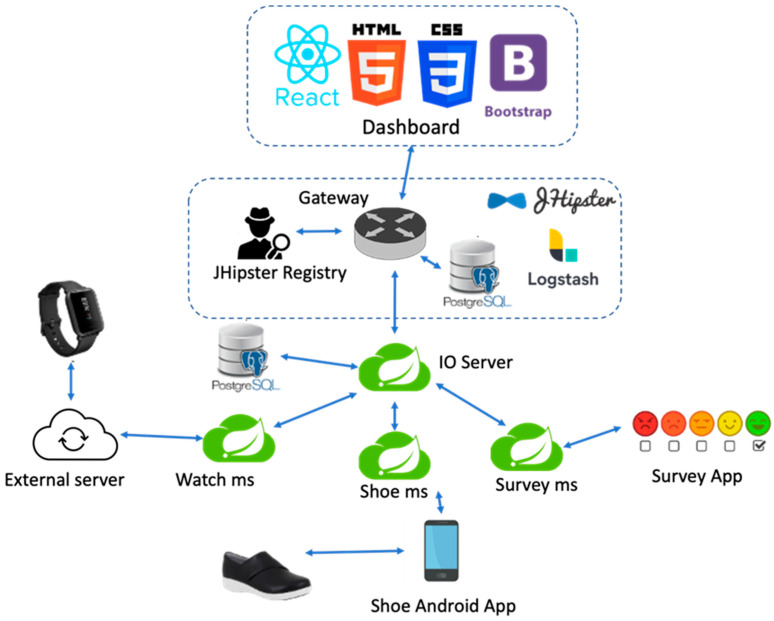
Platform architecture.

The vINCI architecture is based on the JHipster microservices architecture, which provides preconfigured tools and components that offer the management of microservices applications: gateway, JHipster Registry, and JHipster Console. Each of these microservices is responsible for collecting data from the devices and sending the data further to the IO-server microservice [29]. Figure 1 shows the microservices architecture of the vINCI application.

To simplify the schema, the communication links between the JHipster Registry and the rest of the microservices, or between the JHipster console and them, have been removed. However, the gateway and all the newly created microservices still use the JHipster Registry for discovery and the JHipster Console to store logs and metrics.

In the first development phase, the vINCI platform architecture was constituted mainly of the basic JHipster components and the microservices for the CMD watch, Fitbit, insole, and survey microservice.

The architecture was designed so that each microservice had its own PostgreSQL database, where the records from its corresponding device should be stored. The reason was that each device had its own data format and each database would have its specific schema. For example, the watch sends JSON objects that contain coordinates, while the survey application sends JSON objects with fields such as “surveyType” or “scoring Result”.

The gateway is a Java Spring application, like the other microservices generated with JHipster, but with a front-end side written in ReactJS and much more logic of its own. In the case of vINCI, the client uses only one entry for requests: the gateway. It acts as a proxy from client applications to back-end services and vice versa. It exposes a single end point to client applications and maps requests internally to the microservices that should handle the business from that end point. This happens with most microservices architectures, where functionality needs to be used by many services. The API gateway model also allows the developer to more easily add new microservices to the architecture, reduces the overhead of complicated requests, means less duplicate logic and code, and improves security. This task that the gateway performs is called HTTP request routing. It automatically submits each newly added microservice, which can be accessed using its name “/microservice-name”, which is of course secure.

The dashboard is the client side of the gateway microservice. It is an application developed with the latest libraries: ReactJS, bootstrap, HTML5, and CSS3. Since it is written in React, it is very well organized in components, meaning that adding new custom features is very easy. It is built and runs with the npm or yarn JavaScript package managers, which download and install the dependencies needed by the dashboard. The dashboard comes with an administration section for the admin user where one can see information about the microservices that are running in the vINCI platform and their configuration, manage the users, explore application metrics, check the logs, or see the swagger API documentation.

Furthermore, the data saved in IO-Server is fetched and displayed for each device type separately in the entities table. New data can be introduced by the admin manually as well. These are the out-of-the-box features of the gateway. However, more logic has been added to the gateway to satisfy the requirements and objectives of the vINCI project.

When receiving device data, the IO-Server identifies the device from which the information came by a UUID field. However, the information about the device is stored in the tables as an ID, not a string UUID. Therefore, the IO-Server needs to map the received UUID to its corresponding ID from the gateway. The communication between the IO-Server and the gateway is done through REST API calls using a feign client.

The business regarding the watch device is handled by the watch microservice. This is a Spring Boot application. The watches used in the vINCI platform are provided by the Connected Medical Devices company, which developed them to send data periodically to one of its servers (the external server shown in the above figure). For vINCI, the company provided us with an API for importing the data. The watch service makes API calls every 30 s to check if any new data are available. The imported data is then sent to the IO-Server through a feign client, then saved in the specific watch table.

Another important side of the architecture consists of the insole microservice and the insole Android application. The latter allows one or more smart insole devices to connect to it simultaneously and send data to it. The mobile application processes the received data and translates it into more relevant information (i.e., the type of the recorded activity: standing, walking). Then, the information is delivered to the shoe microservice, which forwards it to the IO-Server.

The survey Android application and the survey microservice, written in Spring, handle the business regarding the questionnaires completed by the patients. Unlike the smart shoes or the watches business, the starting point here is not a smart device, but the actual mobile application installed on the patient’s tablet or smart phone. The users complete the surveys, then the answers and the calculated scoring results are sent to the survey microservice as JSON objects. As said before, the specific survey data were stored in a specific PostgreSQL database. 

### 1.4. vINCI Kits

In our study, we used four monitoring kits, but the number can be extended according to the availability and preferences of end users and the monitoring team, and this reflects the flexibility of vINCI technology. The four monitoring kits used in this study were:

- The smart watch (CMD One smart watch) is an existing technology provided by the partner Connected Medical Devices (CMD). The information provided by the smart watch is GPS location/time, the number of steps, how many times the watch has left a defined area, and time intervals when the watch has been removed from the wrist.
- The Fitbit Ionic watch has been integrated into vINCI technology, and is a watch that contains a series of biometric sensors useful in the project for assistive monitoring of the elderly. Fitbit offers users a wide range of physical activity monitoring devices. In the current stage, numerous analyses have been carried out on the validity of these devices in measuring the different indices for adults and the elderly. The Adidas Edition device was selected to offer the main features needed to monitor the elderly. The Fitbit microservice was implemented in Spring Boot (the technology behind the vINCI platform). The tracker communicates with the Fitbit mobile application using BLU technology, which interacts with Fitbit servers using HTTPS.
- Smart insoles, technology developed during the project, which can be used indoors, features a wireless communication interface (BLE or LoRA). The insoles identify different patient activity conditions, namely: standing rest, walking, running, and non-contact (in the sense that shoes are not worn or the foot is not placed on a walking surface). The history of physical activity performed by the subject can be tracked by collecting the data packets transmitted in a given time interval and checking the time stamps applied by the server.
- Questionnaires: WHOQOL-BREF (World Health Organization Quality of Life Questionnaire—Short Form) and IPAQ-SF (International Physical Activity Questionnaire—Short Form). The WHOQOL-BREF was used in the project to measure the quality of life of selected individuals recruited to be part of the study; this questionnaire is available in several languages and for the project, under a legal agreement between us and the World Health Organization, we were granted a license to use the WHOQOL-BREF in accordance with the WHO’s terms and conditions. The WHOQOL-BREF version is a questionnaire with 26 items that assess the quality of life in the physical, psychological, social, and environmental domains. The IPAQ-SF is used to assess the level of physical activity of people recruited to be part of the study; this questionnaire assesses physical activity in many domains, leisure time physical activity, household activities, work-related physical activity, and transport-related physical activity.

The collected data, related to the physical and mental health conditions of the older people, composed a model of the monitored data for each person, which was validated with the support of medical professionals (at the Ana Aslan National Institute of Gerontology and Geriatrics, Bucharest, Romania).

Each vINCI application kit was represented by a microservice. The data collected from patients was medically validated by specialists and then the patient was shown an account of their general condition, receiving recommendations specific to the identified health condition.

All data from surveys and IoT devices was stored in a secure manner to keep data private. This simplified interaction can help detect early symptoms of old age or trigger alerts for special cases such as an older adult’s fall, stroke, etc. This information was provided to the elderly person, their carers, and their family. The patient profile model was used to evaluate the impact of the vINCI technology on the level of quality of life perceived by the elderly adult, allowing an appropriate adjustment of the intervention assistance provided by the family and caregivers [30].

**Figure 2:**
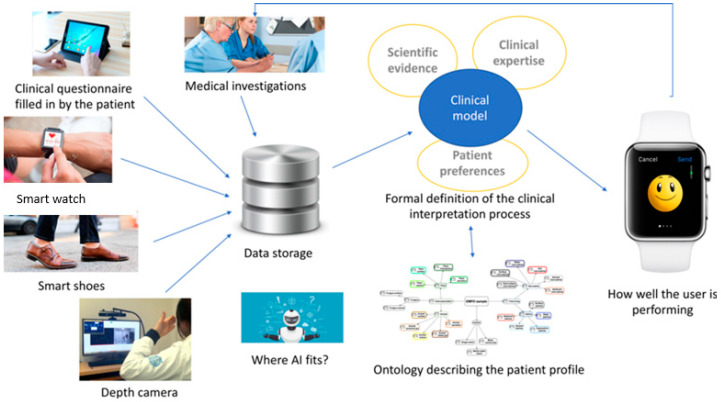
Data flow within the vINCI integrated system.

The data from the devices, derived from information based on a conceptual model, enabled possible alerts based on a detected deterioration in conditions of advanced age, and is shown in Figure 2.

### 1.5. Security and Privacy through Blockchain in vINCI Platform

One of the major problems in health care is the responsibility for data preservation and authenticity. In health care, a variety of personal information is generated by clinics, hospitals, and health-related applications, data that ends up being stored in large databases. During their life, people interact with a large number of medical specialists, for example, pediatricians, general practitioners, nurses, dentists, specialists in medical insurance, etc. Each of them stores data in their IT system, leading to a fragmented system and databases that are not shared.

In our case, the vINCI platform has been developed considering both security and privacy considerations. This means that data being generated for the patient needs to protect their personal data, and access needs to be restricted and fully controlled by the patient alone. Here, some data might be used by clinics, by doctors, or health companies even, but when all these actors need to be involved, who will administer the system? We say that the security of the communicated data needs to be ensured through the use of classic mechanisms, authentication based on certificates, and through the use of blockchain.

In the vINCI platform, the blockchain manages access and permissions to collect health information and use the personal data stored in the platform. For this purpose, the blockchain collaborates with the vINCI platform, the edge nodes, and the Digital Caregiver application. For a starting point in our description, we refer the reader also to our previous paper.

The vINCI blockchain-based data access control system has been developed using the Hyperledger Fabric framework [34], which manages the access and permissions on the information collected, respectively, and the personal data stored in the vINCI platform. 

Blockchain is a digital ledger where all the executed transactions are stored. It uses a distributed peer-to-peer network to make a continuously growing list of ordered records called blocks. Every block contains a set of signed transactions and is validated by the network itself, by means of a consensus mechanism. Copies of the blockchain are distributed on each participating node in the network. Blockchain can be considered as a permanent database because the implemented algorithms prevent alteration of the already stored information. Blockchain provides unified, secure, and user-controlled access to patient health data. It allows users to easily grant, modify, or revoke access to their data.

Blockchain has been integrated as an intermediary in the collaboration between edge nodes and the Digital Caregiver application. The Digital Caregiver application interacts directly with the platform and provides data from patient clinical questionnaire completions and data from medical investigations performed by clinical staff. Entities with the correct permissions can deliver the data to the platform or access health records only when their own identities and cryptographic keys used are verified by the blockchain.

The blockchain structure uses public key cryptography to create an immutable chain of accounts. Each node participating in the network has a copy of the blockchain (tablets and edge nodes, or the vINCI platform). Due to the large amount of data generated, the content of the nodes consists of information links, permissions, and other additional information. The data are either on the participant platform or on the vINCI platform.

**Figure 3:**
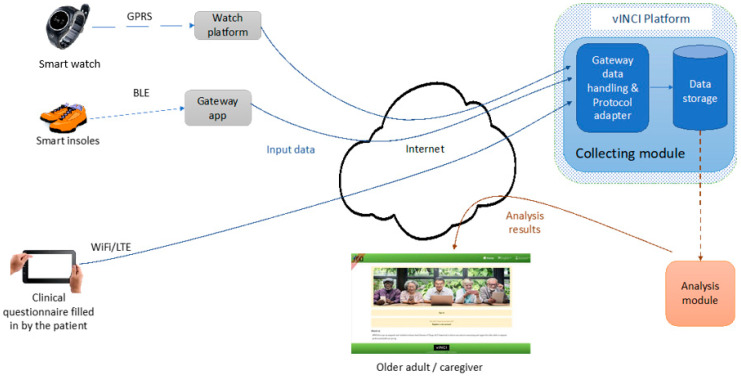
Communication flow.

Clinics, hospitals, research institutes, insurance companies, healthcare applications and other entities that generate medical data, which have the role of nodes, make up the distributed network. Users interact with a particular node and, as a result, data is generated. This stored information is encrypted and digitally signed to ensure the privacy and authenticity of the information. The data must have a certain format accepted by all the other nodes. After this interaction, the node sends a request to the patient asking him/her if the information will be published in the blockchain or not. This communication flow is presented in the Figure 3:

In our implementation, we consider that the proof of work concept is not suitable in healthcare blockchain. Instead, proof of interoperability is an alternative method that eliminates some disadvantages, including powerful and costly hardware required for high computations. Proof of interoperability implies that transactions and stored data are interoperable with regard to a known set of structural and semantic constraints. Structural constraints imply attributes such as type and cardinality, and semantic constraints imply using an agreed value set. Proof of interoperability assumes that all miners reach consensus regarding the set of data templates advised by specialists in medical terminology. The miners, the nodes within a while list, are in fact the medical institutions that check the data format.

**Figure 4:**
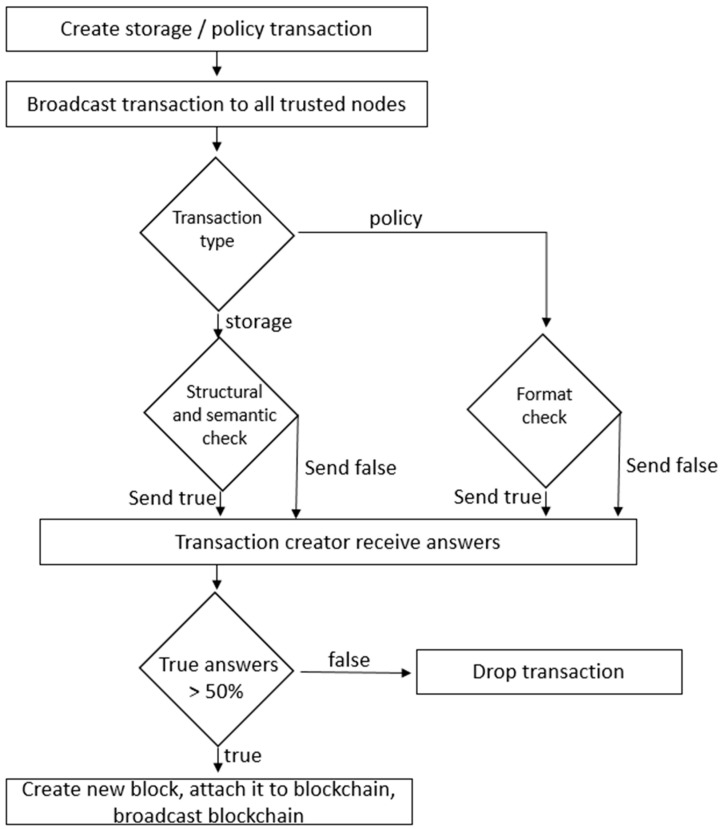
Consensus algorithm.

Therefore, considering the example of the older adult being consulted at a clinic, the institution generates a set of analysis information. This stored information is encrypted and digitally signed to ensure the privacy and authenticity of the information. The institution sends a link with this information and, if the patient agrees to publish it, the clinic broadcasts the transaction to the network. The miners verify the data format and, if the majority agrees, then the clinic generates a new block and appends it to the blockchain in chronological order. All the miners must update it, as shown in the Figure 4.

For vINCI in particular, the blockchain platform implementation, based on open source software—HyperLedger Fabric version 1.4 [35]—includes three modules: microservice blockchain, fabric proxy, and blockchain network. The interaction of the vINCI system with the blockchain network is based on the blockchain microservice and its submodule fabric proxy.

The microservice defines two main entities whose data is saved as transactions in the blockchain network: storage and policy. The storage entity specifies information about a single medical analysis for a patient. A policy entity is defined for each storage transaction and specifies data access policy by calling the appropriate REST API methods defined in the microservice.

The fabric-proxy module is responsible for the integration of the blockchain vINCI microservice with the blockchain network components.

The blockchain microservice is a component of both the vINCI platform and the blockchain platform. Thus, the data saved on the blockchain platform will be reached from the gateway front-end. Validation of both platforms covers the processes of user registration, creation of data access policies, and data requests by the user.

Storage transactions are recorded periodically through REST API communication between analytics modules and the microservice blockchain. These are performed automatically by the analysis module running on the vINCI platform.

The patient defines the list of authorized persons to access personal information through the REST API functions of the blockchain microservice. After this, the policy transaction associated with the storage transaction is saved. At this moment, the person who wants to access the patient data is verified based on the corresponding IDs. The individual queries the appropriate entity for the policy transaction to retrieve the keys to access the patient data.

Based on the security access key received, the patient results saved in the blockchain database can be obtained through the REST API function

## 2. Methodology

### 2.1. Subjects’ Recruitment and Study Design

The validation study of vINCI in Romania was conducted at the Ana Aslan National Institute of Gerontology and Geriatrics (NIGG) Bucharest in 2019–2021 and included two stages: acceptability testing (3 consecutive tests) and clinical validation testing [36]. The clinical validation study was a randomized clinical study that included a convenience sample of older patients admitted to geriatrics wards for various chronic conditions. There was a total number of 60 participants 65 years of age and older, equally divided into two groups, one experimental and one control. The experimental group received the vINCI devices for testing over a period of 7 days while the control group received only usual care, without using the vINCI technology. Each participant in the experimental group was provided with a smart insole, a smart watch, a Fitbit and a smart tablet. The Fitbit device measured the participant’s heart rate, pulse, number of steps taken, and nictemeral rhythm for a period of 7 days. The physical activity level was monitored both subjectively and objectively. The number of steps was counted simultaneously by the Fitbit, the smart watch and the smart insoles. Participants also self-completed the International Physical Activity Questionnaire—Short Form (IPAQ-SF) on the vINCI Tablet using the vINCI application [36]. All the data were integrated into the vINCI application, a flexible platform designed to be able to accept data from various other smart devices.

The inclusion criteria were: people 65 years of age and older living independently in their communities, presence of digital skills, and adequate compliance with the study protocol.

The exclusion criteria were: any acute medical condition, any surgery during the past 3 months, major neurocognitive disorder, moderate and severe depression, visual impairment, angina pectoris, uncontrolled high blood pressure, heart arrhythmias or existing disability that could interfere with functionality, any terminal illness, frailty syndrome, risk of falls, any condition that might limit mobility.

After randomization and group allocation, at the beginning of day 1 of the clinical validation study, each experimental group participant received written and verbal instructions on how to use each device and filled in the WHOQOL-BREF and IPAQ-SF questionnaires on the smart tablet. Technical support was available to the patients if it was needed [36].

The control group received the same questionnaires, but in printed form. On the 8th day of the study, participants in both groups filled in the follow-up WHOQOL-BREF and IPAQ-SF questionnaires, while the participants from the experimental group also filled in the satisfaction questionnaire on the smart tablet.

Before the experiment started, all participants received initial written and verbal instructions about how to use the devices adding details about charging batteries and when and how to wear each device. The researcher designated to ensure the participants understood how to use the vINCI technology verified that each senior was confident about handling and employing the devices on the first day of the experiment and before the actual data collection. Written instructions were handed over to the users so that they could refer to them at any time during the experiment. The participants were told that the researcher was easily reachable during the duration of the experiment in case they needed any other guidance or in case there were any technical issues to be addressed. None of the subjects requested any additional assistance with the devices over the 7 consecutive testing days.

### 2.2. Data Collection

#### 2.2.1. Quality of Life Assessment (WHOQOL-BREF)

The quality of life (QoL) questionnaire used in this study was WHOQOL-BREF, the Romanian version. The WHOQOL-BREF is a standardized questionnaire that includes 24 assessment items grouped in four major categories: physical health, psychological health, social relationships, and environmental health. It also includes 2 separate questions about individual’s overall perception of quality of life and their health. Response choices are organized on a 5-level incremental scale that estimates perception over the past 2 weeks. The raw scores were converted to transformed scores on a scale of 4–20 and domain scores were transformed to a 0–100 scale; the higher the score, the higher the health-related quality of life. A good QoL is indicated by a score of at least 60 points, while a lower score points toward a compromised QoL, as specified in other studies [36,37].

#### 2.2.2. Physical Activity Assessment (IPAQ-SF)

An optimum physical activity (PA) level is a prerequisite for a healthy lifestyle, an active longevity with a preserved functional independence and a good quality of life [38]. It is well known that a sedentary lifestyle and a low physical activity level are major risk factors for many non-communicable diseases such as cardiovascular diseases, diabetes, osteoporosis and many others. On the other hand, enhancing physical activity, especially in older age, has many recognized health benefits and a positive impact of the overall quality of life and well-being. PA levels can be assessed with many different instruments, either subjectively with questionnaires and interviews or objectively using various sensors and monitors [39,40].

The IPAQ-SF questionnaire used in the present study asks about three specific types of activity undertaken over a previous period of one week: walking activity, PA of moderate intensity, and PA of vigorous intensity [41]. MET minutes represent the amount of energy expended carrying out physical activity. A MET is a multiple of one’s estimated resting energy expenditure.

The scoring of the IPAQ-SF questionnaire allows identification of low, moderate, and high levels of PA [41,42]. A high level of PA was considered when respondents had engaged in vigorous activities for at least 3 days during the previous week (>1500 MET-minutes/week) or in any specific combination of exemplified activities (or >3000 MET-minutes/week). A moderate level of PA was defined as engaging in at least half an hour of moderate-intensity activities most days of the week (600 to 1499 MET-minutes/week). A low level of PA means the respondents did not participate in any regular daily PA and did not meet the criteria for moderate or high levels of PA.

### 2.3. Data Analysis and Processing

Statistical analysis was executed with the IBM SPSS Statistics 24 software. The sociodemographic characteristics of the sample were depicted by the mean and standard deviation or proportion, as appropriate.

In the first step, we used the SPSS syntax file [43] that automatically checks and recodes data and computes domain scores for scales in the range 4–20. Then, we used a SPSS syntax file developed by the vINCI team to calculate the domain scores for scales in the range 0–100. We then analyzed the data from the samples both at the level of each item and at the level of each WHOQOL-BREF domain [44,45]. Descriptive statistics were calculated and reported (e.g., mean, standard deviation, median) and the Shapiro–Wilk normality test was performed. Construct validity was evaluated by domain-to-domain correlations and by correlating item Q1 (Overall QoL) and item Q2 (General health) with the four domains in the WHOQOL-BREF using the Pearson correlation coefficient.

The instructions of the November 2005 version of the guidelines for data processing and analysis of the IPAQ-SF were used for data cleaning and processing prior to computing the algorithms [41,46]. The present study applied rules 1 to 6 from the specific guideline [47]. 

## 3. Results

### 3.1. Sample Characteristics—Control and Experimental Groups

As Table 1 depicts, the sample was balanced for gender. Moreover, the age, marital status, educational level, and health status did not show major differences between the control and experimental groups. Therefore, it can be concluded that the two groups were comparable.

### 3.2. Results—Control Group (Day 1 and Day 8)

#### 3.2.1. WHOQOL-BREF

##### Item-Level Analysis

In the control group (day 1) items Q23 (home environment), Q24 (access to health care), Q13 (daily information), and Q20 (personal relationship) were the four highest-scoring items (written in the scores’ descending order). On the other hand, Q4 (dependence medication) and Q16 (sleep and rest) scored lowest among the 24 items. The mean scores ± standard deviation (SD) for the other two questions, Q1 and Q2, were 4.13 and 3.57. These questions should be evaluated separately and are not included in a domain, as per the WHOQOL-BREF manual [44,45].

In the control group (day 8) items Q23 (home environment), Q20 (personal relationship), Q13 (daily information), and Q24 (access to health care) were the four highest-scoring items (written in the scores’ descending order). On the other hand, Q4 (dependence medication) and Q16 (sleep and rest) scored lowest among the 24 items. The mean scores ± SD for the other two questions, Q1 and Q2, were 4.20 and 3.90.

**Figure 5:**
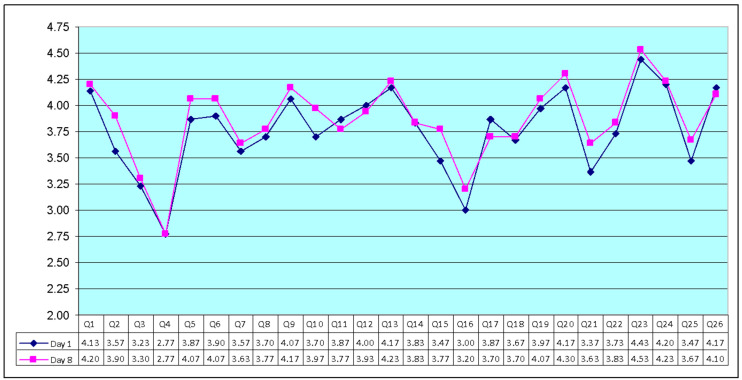
The mean scores of the 26 items in WHOQOL-BREF scale (control group, day 1 and day 8, N = 30).

Figure 5 shows the average of the items on day 1 and day 8. There was no significant difference in scores between day 1 and day 8 (the results are not shown).

##### Domain-Level Analysis

The mean scores ± SD for the four domains of the WHOQOL-BREF, in the transformed scores of 0–100, are shown in Table 2 (day 1 and day 8). When comparing the four domains, the environmental domain was the highest with a mean score of 74.58 (day 1) and 76.12 (day 8), while the physical domain was the lowest (59.64 and 62.15, respectively).

A total score below 60 points signals impaired QoL (social domain in day 1) and a score of at least 60 points identifies patients with a good QoL (all other domains on day 1 and day 8).

By using Pearson correlation coefficient, it was possible to establish the link between general health, overall QoL, and the four domains. Results are shown in Table 3 (day 1 and day 8).

A high positive correlation between the physical and psychological domains, with high levels of physical domain associated with higher levels of psychological domain (r = 0.65, *p* < 0.01), was established on day 1 using Pearson correlation coefficient. Overall QoL (Q1) was positively interrelated with all the other four domains, but significantly only with the psychological domain (moderate correlation, r = 0.39, *p* < 0.05). The four domains and general health (Q2) were significantly and positively interrelated with moderate to high relationships, ranging from 0.43 (*p* < 0.05) to 0.58 (*p* < 0.01), except for the environment domain (Table 3).

On day 8, the Pearson correlation coefficient showed a significantly high positive correlation between the physical and psychological domains (r = 0.69, *p* < 0.01), between the psychological and social domains (r = 0.52, *p* < 0.01) and between the psychological and environmental domains (r = 0.50, *p* < 0.01). There was also a significant, positive, and moderate correlation between the physical and social domains (r = 0.48, *p* < 0.01). The four domains and overall QoL (Q1) were significantly and positively interrelated with moderate to high relationships, ranging from 0.42 (*p* < 0.05) to 0.57 (*p* < 0.01), except for the physical domain (Table 2). General health (Q2) was positively related to all four domains, but significantly only with the physical domain (high, r = 0.57, *p* < 0.01) and the social domain (moderate, r = 0.40, *p* < 0.05) (Table 3).

#### 3.2.2. IPAQ-SF

This study used the same Romanian control sample of patients (day 1 and day 8, N = 30) from whom data were collected for the WHOQOL-BREF instrument [48]. Table 4 shows the IPAQ-SF final results.

In the control group (day 1), of the 30 patients, 33.3% had a high level of PA, 53.5% had a moderate level of PA, and 13.3% had a low level of PA. The median value of the total physical activity (MET-minutes/ week) was 1801.50.

In the control group (day 8), of the 30 patients, 43.3% had a high level of PA, 50.0% had a moderate level of PA, and 6.7% had a low level of PA. The median value of the total physical activity (MET-minutes/ week) was 2029.50.

### 3.3. Results—Experimental Group (Day 1 and Day 8)

#### 3.3.1. WHOQOL-BREF

##### Item-Level Analysis

In the experimental group (day 1) items Q23 (home environment), Q13 (daily information), Q9 (physical environment), and Q20 (personal relationship), were the four highest-scoring items (written in the scores’ descending order). On the other hand, Q4 (dependence medication) and Q3 (physical pain) scored lowest among the 24 items. The mean scores ± SD for the other two questions, Q1 and Q2, were 3.97 and 3.53. These questions should be evaluated separately and are not included in a domain, as per the WHOQOL-BREF manual [44,45].

In the experimental group (day 8), items Q23 (home environment), Q13 (daily information), Q20 (personal relationship), and Q24 (access to health care) were the four highest-scoring items (written in the scores’ descending order). On the other hand, Q4 (dependence medication) and Q25 (transport) scored lowest among the 24 items. The mean scores ± SD for the other two questions, Q1 and Q2, were 4.27 and 3.77, respectively.

**Figure 6:**
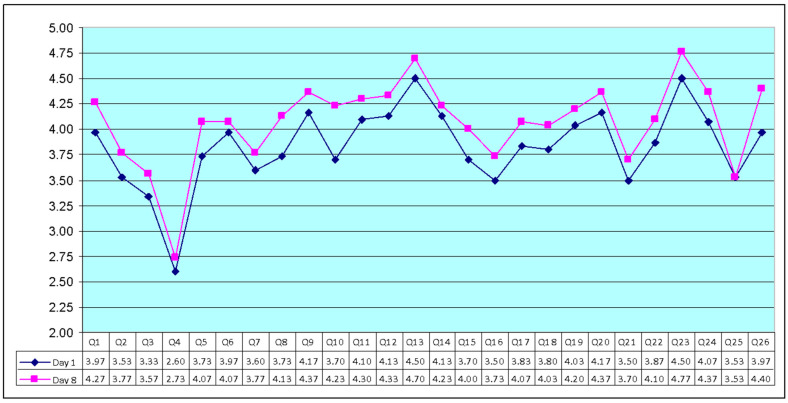
The mean scores of the 26 items in WHOQOL-BREF scale (experimental group, day 1 and day 8, N = 30).

Figure 6 shows the average of the items on day 1 and day 8. In the experimental group, the results showed that there were statistically significant differences between day 1 and day 8 regarding quality of life (QoL).

##### Domain-Level Analysis

The mean scores ± SD for the four domains of the WHOQOL-BREF, in the transformed scores of 0–100, are shown in Table 5. Comparing the four domains, the environmental domain was the highest with a mean score of 82.60 (day 8) and 77.40 (day 1), while the physical domain was the lowest (62.38 in day 1 and 69.17 in day 8).

A score of at least 60 points identifies patients with a good QoL (all the domains on day 1 and day 8).

The relationship between the four domains, overall QoL and general health was investigated using Pearson product-moment correlation coefficients. Results are shown in Table 6 (day 1 and day 8).

A high positive correlation between the four QoL domains, ranging from 0.53 (*p* < 0.01) to 0.62 (*p* < 0.01), except for the correlation between the physical and social domains, was established on the first day using the Pearson correlation coefficient. The four domains and overall QoL (Q1) were significantly and positively interrelated with moderate to high relationships, ranging from 0.39 (*p* < 0.05) to 0.61 (*p* < 0.01), except for the physical domain (Table 6). General health (Q2) was significantly and highly positively interrelated with all the four domains, ranging from 0.56 (*p* < 0.01) to 0.69 (*p* < 0.01). There was a significant and moderate positive correlation between the overall QoL and general health (r = 0.40, *p* < 0.01).

On the eighth day, the Pearson correlation coefficient showed a significant and positive correlation between the four QoL domains, ranging from moderate 0.48 (*p* < 0.01) to high 0.68 (*p* < 0.01), except for the correlation between the physical and social domains. The four domains and overall QoL (Q1) were significantly and positively interrelated with moderate to high relationships, ranging from 0.37 (*p* < 0.05) to 0.61 (*p* < 0.01), except for the environmental domain (Table 6). General health (Q2) was significantly and positively interrelated with the four domains with moderate to high relationships, ranging from 0.43 (*p* < 0.05) to 0.71 (*p* < 0.01), except for the environmental domain. There was a significant and high positive correlation between the overall QoL and general health (r = 0.55, *p* < 0.01) (Table 6).

#### 3.3.2. IPAQ-SF

This study used the same Romanian experimental sample of patients (day 1 and day 8, N = 30) from whom data were collected for the WHOQOL-BREF instrument [48]. Table 7 shows the IPAQ-SF final results.

In the experimental group (day 1), of the 30 patients, 63.3% had a high level of PA, 33.4% had a moderate level of PA, and 3.3% had a low level of PA. The median value of total PA (MET-minutes/week) was 3066.00.

In the experimental group (day 8), of the 30 patients, 66.7% had a high level of PA, 33.4% had a moderate level of PA, and 0.0% had a low level of PA. The median value of total PA (MET-minutes/week) was 3304.50.

#### 3.3.3. vINCI Technology—Satisfaction Questionnaire

One questionnaire was developed (in line with the objectives of vINCI project) in order to measure the degree of user satisfaction regarding the use of vINCI technology and services [49].

The users’ answers regarding the frequency of use of the vINCI application, as well as the distribution of the answers according to gender, were analyzed. Participants had the opportunity to indicate on a scale from 1 to 5 how often they used the vINCI application. The proposed scale measure has the following significance:

1. Daily: study participants used the vINCI app daily. The impact of these users is high;
2. Weekly: study participants used the vINCI application once or several times a week;
3. Monthly: study participants used the vINCI application once or several times a month;
4. Several times a year: study participants used the vINCI application once or several times a year;
5. Never: users did not use the vINCI application.

Table 8 presents information regarding the answers provided by the older adults.

According to the satisfaction questionnaire (Table 8), the study’s participants considered the vINCI devices easy to use and understand (e.g., Q13: “The interaction with the vINCI application is clear and easy to understand”, *M* = 4.07, *SD* = 0.63), without a negative interference on their daily activities (e.g., Q11: “The daily monitoring performed through the vINCI application does not interfere with my personal data”, *M* = 4.20, *SD* = 0.61).

## 4. Discussion

During the past decade, technological assisted living for healthy aging has become a distinct research field with many initiatives. Using monitoring sensors, other projects developed intelligent home care systems, step counters, location, and identification systems, signal algorithms for analyzing heart arrhythmias and cognitive function promotion systems [50,51,52,53].

There are several original aspects of our project that we would like to highlight.

Firstly, the vINCI technology is a tripod system with a user-friendly interface in the form of a smart phone application specifically designed for older people, an integrative platform that allows networking as many sensors as necessary to provide different models adapted to the care needs of older people and dedicated sensors for physical activity and location monitoring.

Secondly, the physical activity level is evaluated both subjectively and objectively, providing an accurate estimation, while the feedback received by the user is based on a medical backed algorithm.

Another original feature of our project is that testing of the vINCI technology was in clinical settings, in a controlled environment on actual older patients with various chronic diseases.

As presented, the results of the clinical study are promising. The patients admitted to the Ana Aslan NIGG clinics showed a high interest in the use of smart devices and in completing the experiment and the attrition rate was 0%.

Participants were open and motivated to wear these devices to obtain specific information about a range of biophysiological measurements. The use of Fitbit raised particular interest by providing easily accessible additional information.

According to the satisfaction questionnaire, the study’s participants considered the vINCI devices easy to use and understand, without a negative interference on their daily activities. The older patients were generally satisfied with the use of vINCI devices for intelligent continuous monitoring of clinical status and behavior.

In the experimental group, the results showed that there were statistically significant differences between day 1 and day 8 regarding quality of life (QoL) in each of the WHOQOL-BREF domains: physical, psychological, social, and environmental. Finally, in the control group, the results showed that there were no statistically significant differences in scores for participants between day 1 and day 8 regarding QoL in each of the WHOQOL-BREF domains, except for the social domain.

In the experimental group (day 8), of the 30 patients, 66.7% had a high level of physical activity (PA) and the median value of total PA (MET-minutes/week) was 3304.50, the high category describing high levels of PA participation. In the control group (day 8), of the 30 patients, 50.0% had a moderate level of PA and the median value of total PA (MET-minutes/week) was 2029.50.

We also identified several limitations of our clinical pilot study.

The sample size was small and the duration of the intervention was short, although these features were characteristic for a pilot study.

Secondly, we can speculate that the significant increase in the physical activity levels and health-related quality of life might have been partly due to a confounding motivation of the participants generated by the attention they received from our supporting staff during the experiment.

## 5. Conclusions

vINCI developed a flexible, adaptive, and older people-friendly active monitoring technology that provides care-related feedback and enables users to adjust their lifestyles in order to achieve an overall better health-related quality of life. As demonstrated in our previous work, the technology can successfully be independently used by older adults due to its user friendly interface and low level of intrusion in their daily lives.

In this work, the impact of the technology on the quality of life of patients in the experimental and control groups recruited by NIGG was highlighted.

The results of the statistical analysis of the collected data showed that there were statistically significant differences between day 1 and day 8 in quality of life (QoL) in each of the WHOQOL-BREF domains: physical, psychological, social, and environmental, the period in which these technologies were used. Additionally, another important result highlighted was related to user satisfaction after using the vINCI technology.

According to the results obtained from the experiment carried out by NIGG, it appears that the technologies developed and delivered in the vINCI project bring real benefits to the users. This study could be of real use to companies and researchers developing smart health products for specific IT systems.

Future studies on larger samples and in various settings are needed in order to better define and understand the optimal vINCI system models for older adults’ healthcare needs.

## Figures and Tables

**Table 1:** Sample characteristics for the control (N = 30) and experimental (N = 30) groups.

		Control	Experimental
Gen	Male	15	15
	Female	15	15
Age	Mean (SD)	72.50 (6.09)	71.40 (5.92)
	Range	65–85	65–85
Marital status *n* (%)	Single	1 (3.3)	0 (0.0)
	Married	17 (56.7)	19 (63.3)
	Divorced	2 (6.7)	3 (10.0)
	Living as married	1 (3.3)	1 (3.3)
	Widowed	9 (30.0)	7 (23.3)
Education *n* (%)	Primary	0 (0.0)	2 (6.7)
	Secondary	23 (76.7)	19 (63.3)
	Tertiary or higher	7 (23.3)	9 (30.0)
Health status *n* (%)	Healthy	10 (33.3)	7 (23.3)
	Unhealthy	20 (66.7)	23 (76.7)

Note. The participant information was controlled by the researchers so that it is the same for day 1 and day 8.

**Table 2:** Domains: summary statistics (transformed scores 0–100; control group, day 1 and day 8, N = 30).

Domain	Day 1			Day 8		
	Mean	SD	Median	Mean	SD	Median
Physical	59.64	16.78	62.50	62.15	18.98	64.29
Psychological	72.22	11.34	72.92	73.75	11.69	75.00
Social	68.89	9.00	66.67	73.06	12.70	75.00
Environmental	74.58	11.00	75.00	76.12	10.13	78.12

**Table 3:** Pearson correlations of the four QoL domains, overall QoL and general health (control group, day 1 and day 8, N = 30).

	Physical	Psychological	Social	Environmental	Overall QoL	General Health
**Day 1**						
Physical	1.0	0.65 **	0.33	0.28	0.29	0.58 **
Psychological		1.0	0.26	0.14	0.39 *	0.52 **
Social			1.0	0.06	0.31	0.43 *
Environmental				1.0	0.34	0.26
Overall QoL (Q1)					1.0	0.53 **
General health (Q2)						1.0
**Day 8**						
Physical	1.0	0.69 **	0.48 **	0.29	0.10	0.57 **
Psychological		1.0	0.52 **	0.50 **	0.52 **	0.21
Social			1.0	0.34	0.57 **	0.40 *
Environmental				1.0	0.42 *	0.22
Overall QoL (Q1)					1.0	−0.08
General health (Q2)						1.0

* *p* < 0.05; ** *p* < 0.01.

**Table 4:** IPAQ-SF MET-minutes/week and continuous scores (control group, day 1 and day 8, N = 30).

Physical Activity (PA)	Day 1			Day 8		
	Median	Percentiles		Median	Percentiles	
		25	75		25	75
Vigorous (MET-minutes/week)	0.00	0.00	0.00	0.00	0.00	0.00
Moderate (MET-minutes/week)	410.00	0.00	960.00	490.00	195.00	1020.00
Walking (MET-minutes/week)	1386.00	486.75	2252.25	1435.50	693.00	2772.00
Total PA (MET-minutes/week)	1801.50	1055.25	3562.50	2029.50	1082.25	4025.50

**Table 5:** Domains: summary statistics (transformed scores 0–100; experimental group, day 1 and day 8, N = 30).

Domain	Day 1			Day 8		
	Mean	SD	Median	Mean	SD	Median
Physical	62.38	18.78	60.71	69.17	20.26	69.64
Psychological	72.50	13.16	70.83	78.33	12.64	79.17
Social	71.11	15.43	75.00	76.39	12.96	75.00
Environmental	77.40	9.97	78.13	82.60	9.42	79.69

**Table 6:** Pearson correlations of the four QOL domains, overall QOL and general health (experimental group, day 1 and day 8, N = 30).

	Physical	Psychological	Social	Environmental	Overall QOL	General Health
**Day 1**						
Physical	1.0	0.56 **	0.34	0.56 **	0.06	0.56 **
Psychological		1.0	0.53 **	0.56 **	0.39 *	0.67 **
Social			1.0	0.62 **	0.56 **	0.69 **
Environmental				1.0	0.61 **	0.68 **
Overall QoL (Q1)					1.0	0.40 *
General health (Q2)						1.0
**Day 8**						
Physical	1.0	0.63 **	0.35	0.48 **	0.43 **	0.43 *
Psychological		1.0	0.59 **	0.68 **	0.61 **	0.71 **
Social			1.0	0.48 **	0.37 *	0.57 **
Environmental				1.0	0.30	0.30
Overall QoL (Q1)					1.0	0.55 **
General health (Q2)						1.0

* *p* < 0.05; ** *p* < 0.01.

**Table 7:** IPAQ-SF MET-minutes/week and continuous scores (experimental group, day 1 and day 8, N = 30).

Physical Activity (PA)	Day 1			Day 8		
	Median	Percentiles		Median	Percentiles	
		25	75		25	75
Vigorous (MET-minutes/week)	0.00	0.00	240.00	0.00	0.00	240.00
Moderate (MET-minutes/week)	840.00	280.00	1860.00	840.00	375.00	2100.00
Walking (MET-minutes/week)	1386.00	839.03	2772.00	2079.00	1386.00	3093.75
Total (MET-minutes/week)	3066.00	1747.00	4652.25	3304.50	2227.50	5005.00

**Table 8:** Descriptive statistics about the satisfaction of the older adults with vINCI technology.

Item	Question	Mean	Std. Deviation
Q1	“It is easy to learn how to work with the vINCI application”	4.07	0.69
Q2	“The vINCI application is easy to use”	4.07	0.76
Q3	“Using the vINCI app, I am better informed about my health”	3.93	0.76
Q4	“My security level has improved using the vINCI application”	3.53	0.68
Q5	“The vINCI application helps me to obtain relevant quality of life data”	3.82	0.91
Q6	“The vINCI application gives me the opportunity to more easily communicate data about my physical condition/quality of life”	3.90	0.75
Q7	“The system interface is pleasant and intuitive”	3.95	0.77
Q8	“The results provided by the application are easy to access and understand”	3.75	0.63
Q9	“I think I could improve my health using the vINCI app”	3.78	0.81
Q10	“The information provided by the vINCI application is complete and useful”	3.71	0.52
Q11	“The daily monitoring performed through the vINCI application does not interfere with my personal data”	4.20	0.61
Q12	“The vINCI application has improved the quality of medical services received”	3.82	0.85
Q13	“The interaction with the vINCI application is clear and easy to understand”	4.07	0.63
Q14	“The organization of the information on the screen of the devices running the vINCI application is clear and intuitive”	3.93	0.55
Q15	“The vINCI application is very useful for me in my daily life”	3.67	0.90
Q16	“Using the vINCI application is very exciting”	3.70	0.74
Q17	“I like to interact with the vINCI application interface”	3.80	0.68
Q18	“I use the vINCI application with confidence”	3.88	0.67
Q19	“Overall, I am satisfied with how to use the vINCI application”	3.93	0.61

## Data Availability

The dataset used and analyzed during the current study is available from the corresponding author on reasonable request.

## Abbreviations

API	Application Programming Interface
BLE	Bluetooth Low Energy
CSS3	Cascading Style Sheets, a declarative stylesheet language for structured documents
HTML	Hypertext Markup Language
HTTP	Hypertext Transfer Protocol
IoT	Internet of Things
IPAO	International Physical Activity Questionnaire
JHipster	Java Hipster—a free and open-source application generator used to quickly modern web applications
JSON	JavaScript Object Notation
LoRA	Long Range
LTE	Long-Term Evolution
NIGG	National Institute of Gerontology and Geriatrics
PA	Physical Activity
PostgreSQL	Postgres is a free and open-source relational database management system (RDBMS)
QoL	Quality of Life
ReactJS	A free and open-source front-end JavaScript library
SD	Standard deviation
UUID	Universally Unique Identifier
vINCI	Clinically-validated INtegrated Support for Assistive Care and Lifestyle Improvement: the Human Link
WHO	World Health Organization
WHOQOL-BREF	World Health Organization Quality of Life Questionnaire—Short Form
